# Self-Reference Refractive Index Sensor Based on Independently Controlled Double Resonances in Side-Coupled U-Shaped Resonators

**DOI:** 10.3390/s18051376

**Published:** 2018-04-28

**Authors:** Xiaobin Ren, Kun Ren, Chengguo Ming

**Affiliations:** 1 School of Science, Tianjin University of Science and Technology, Tianjin 300222, China; renxiaobin@tust.edu.cn (X.R.); mingchengguo@tust.edu.cn (C.M.); 2 College of Precision Instrument and Opto-Electronics Engineering, Key Laboratory of Opto-Electronics Information Technology (Tianjin University), Ministry of Education, Tianjin 300072, China

**Keywords:** refractive index sensor, plasmon-induced transparency, surface plasmon polaritons, plasmonic waveguide

## Abstract

A plasmonic, refractive, index nanosensor is investigated theoretically and numerically in two U-shaped cavities side-coupled to a metal–dielectric–metal (MDM) waveguide. A transparency window between two transmission dips is observed. The physical origin of the transmission phenomenon is revealed by mapping the magnetic field distribution. Independent double resonances are realized through the proposed design. Double resonances showed diverse responses to the variations of the structural dimensions. In particular, they presented different dependences on a refraction index of the medium in an individual resonator. One resonance exhibited a remarkable shift with the increase of the refraction index; however, the other resonance remained unchanged. On the basis of this unique characteristic of differing sensitivities, self-reference sensing is discussed. The nanosensor yielded a high sensitivity of 917 nm/RIU and a figure of merit of 180 RIU^−1^. This work is helpful in terms of the design of on-chip optical sensors with high sensitivity and improved detection accuracy in complicated environments.

## 1. Introduction

Refractive index sensors with high sensitivity are highly valuable, with important applications in disease diagnostics, biology, environmental monitoring, etc. Refractive index sensors based on surface plasmon polaritons (SPPs) have attracted considerable attention [1,2]. SPPs are formed by interactions between incident photons and free electrons on metal surfaces. They can overcome classical diffraction limit and reduce the size of components to nanoscale [3,4]. As a result of the strong enhancement of mode field and the high confinement to the interface of a metal insulator, SPPs are very sensitive to the variation of surrounding materials. Plasmonic sensors have been investigated and developed, such as temperature sensors [5] and biosensors [6,7].

In comparison with array plasmonic structures, metal–dielectric–metal (MDM) waveguides are more compact and are fabricated more easily. They are good candidates for integrated optical circuits. A highly sensitive nanosensor with a sensitivity of 900 nm/RIU was achieved in a plasmonic waveguide side-coupled with a pair of nanoresonators [8]. On the basis of Fano resonances, a single defect nanocavity coupled with a plasmonic waveguide achieved a sensitivity of 700 nm/RIU [9]. An MDM waveguide with two silver baffles and a coupled ring cavity yielded a sensitivity of 718 nm/RIU [10]. A refractive index sensor with a figure of merit of 75 RIU^−1^ was obtained using Fano resonance in a MDM waveguide coupled with resonators [11]. Dynamically tunable resonance also was investigated based on a graphene, nanocavity-coupled, waveguide system; the plasmonic refractive index sensor achieved a sensibility of 333 nm/RIU [12].

With the improvement of sensitivity, an unstable environment, such as temperature fluctuation, can shift the resonant wavelength and, accordingly, affect measurement accuracy. To guarantee detection accuracy in a complicated environment, a sensor with self-reference performance is proposed in which independent multiple resonances are required. It has been reported that multiple resonances have different responses to variations in geometric parameters [13,14,15]. However, all resonant wavelengths are sensitive to a variation of refractive index. Recently, independent double Fano resonances were demonstrated in a simple metallic grating structure [16]. These Fano resonances had significantly different sensitivities to the refractive index variations of the medium above the metal surface. The different sensitivities to index variation were utilized to realize the self-reference sensor. The obtained sensitivity and figure of merit was 470 nm/RIU and 31 RIU^−1^, respectively [16].

In this paper, we investigate independent multiple resonances in the MDM waveguide system. The transmission spectrum and magnetic field distributions were investigated using the finite element method (FEM) of COMSOL Multiphysics. The formation mechanism of the resonances was analyzed by the temporal coupled-mode theory (CMT). The influences of the structural parameters and index of refraction on the transmission characteristics are analyzed. A simple self-reference sensor with high sensitivity is demonstrated.

## 2. The Sensing Structure and Theoretical Analysis

**Figure 1:**
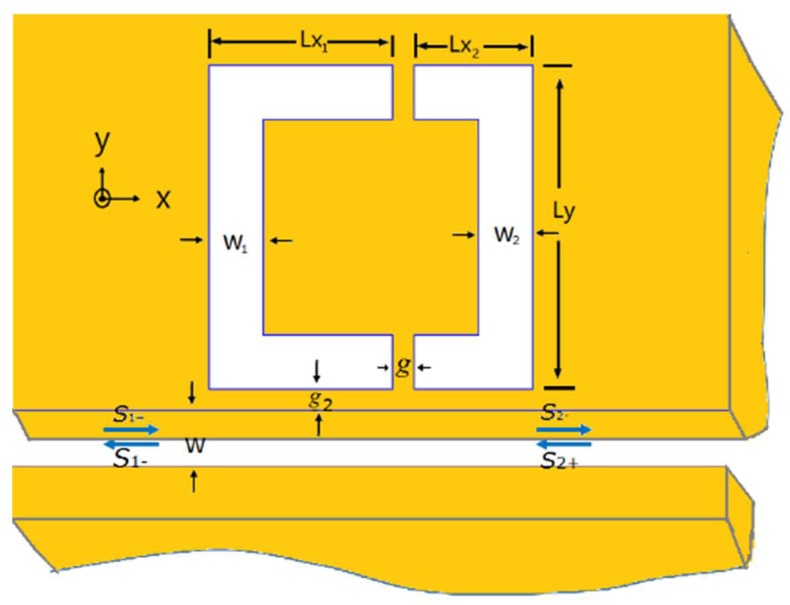
Schematic diagram of U-shaped nanocavity side-coupled to a MDM waveguide. The width of waveguide is *W*. The width of left and right cavity is *W*_1_ and *W*_2_. *Lx* and *Ly* describe the length of cavities along *x*- and *y*-direction, respectively. The gap between the two cavities is *g*.

The proposed MDM nanostructure is schematically shown in Figure 1. Two U-shaped resonators were side-coupled to a plasmonic waveguide. The coupling distance between resonators and waveguide is *g*_2_. The width of MDM waveguide is *W*. The width of left and right U-shaped resonator is *W*_1_ and *W*_2_, respectively. *Ly* denotes the length of U-resonators along *y*-direction. The arm lengths of resonators along *x*-direction are given by *Lx*_1_ and *Lx*_2_, respectively. The gap between the two U-resonators is *g*. Dielectric (*ε_d_*) and metal (*ε_m_*) is denoted by white and yellow, respectively. The background metal was chosen to be silver, the complex relative permittivity of which is characterized by the well-known Drude model: $\varepsilon_{m}=\varepsilon_{\infty }-\omega_{p}^{2}/\left(\omega^{2}+i\gamma \omega \right)$, where *ε*_∞_ = 3.7 is the dielectric constant at infinite angular frequency, *ω* stands for the angular frequency of the incident light, *ω_p_* = 1.38 × 10^16^ Hz is the bulk plasma frequency, and *γ* = 2.73 × 10^13^ Hz is the damping frequency of the oscillations.

For the waveguide coupled U-resonator, the resonance modes satisfy the standing wave condition. The resonance wavelengths are determined by:

*λ*_m_ = 2Re(*n*_eff_)*L*/(*m − φ*_r_/*π*), (1)

where *L* is the length of resonator, *m* is an integer, and *φ*_r_ is the phase shift caused by the reflection on the end of the resonator. Re(*n*_eff_) is the real part of effective index for SPPs in the resonator and is given by *n*_eff_ = *β*/*k*_0_, where *β* is the propagation constant of the SPP. *β* can be obtained from the dispersion equation of the TM mode in the waveguide [17,18]: *ε_d_ k_d_* + *ε_m_k_d_* tanh (*k_d_W*/2) = 0, where *W* is the width of waveguide. *k_d_* and *k_d_* are transverse propagation constants in the dielectric and the metal, respectively. $k_{d}=\sqrt{\beta^{2}-k_{0}^{2}\varepsilon_{d}}$, $k_{m}=\sqrt{\beta^{2}-k_{0}^{2}\varepsilon_{m\;}}$, where *k*_0_ represents the wave number of light in free space.

When optical waves were launched only from the input port of the bus waveguide, *S*_2+_ = 0. Using the coupled mode theory (CMT) [19,20], for two U-shaped resonators side-coupled to the MDM waveguide, the time evolution of the energy amplitudes *a*_1_ and *a*_2_ for two resonators can be described by:

(2) $$\frac{da}{dt}=\left(j\omega_{1}-\kappa_{0}\right)a+j\sqrt{\kappa_{1}}b$$

(3) $$\frac{db}{dt}=\left(j\omega_{2}-\kappa_{0}\right)b+j\sqrt{\kappa_{1}}a$$

where *κ*_0_ is the decay rate due to the internal loss in the cavity, *κ*_1_ are coupling coefficients related to the inter-space. *ω*_1_ and *ω*_2_ are resonant frequencies of two independent resonators. To simplify the model, we ignore the influence of *κ*_0_. Then, the resonant frequencies of the coupled system *ω*_+_ and *ω*_−_ can be deduced as:

(4) $$\omega_{\pm }=\frac{\omega_{1}+\omega_{2}}{2}\pm \sqrt{{\left(\frac{\omega_{1}-\omega_{2}}{2}\right)}^{2}+{\left|\kappa_{2}\right|}^{2}}=\frac{\omega_{1}+\omega_{2}}{2}\pm \Omega_{0}$$

If the two resonators have the same dimension, then *ω*_1_ = *ω*_2_, the resonant frequency *ω*_1_ will split into *ω*_+_ and *ω*_−_ because of the destructive interference between two resonators. The transmission spectra of the two coupled resonators would exhibit two transmission dips. The bandwidth of transparency window between the coupled modes can be expressed as Δ*ω* = 2Ω_0_ = 2|*κ*_2_|.

To investigate the spectral responses of the proposed structure, numerical simulations were performed by COMSOL Multiphysics. In the simulations, we fixed *W* = 50 nm, *g*_2_ = 20 nm and *Ly* = 300 nm. The initial dielectric filled in the U-shaped resonators and waveguide was air, i.e., *ε_d_* = 1.

## 3. Results and Discussion

### 3.1. Transmission Characteristics

**Figure 2:**
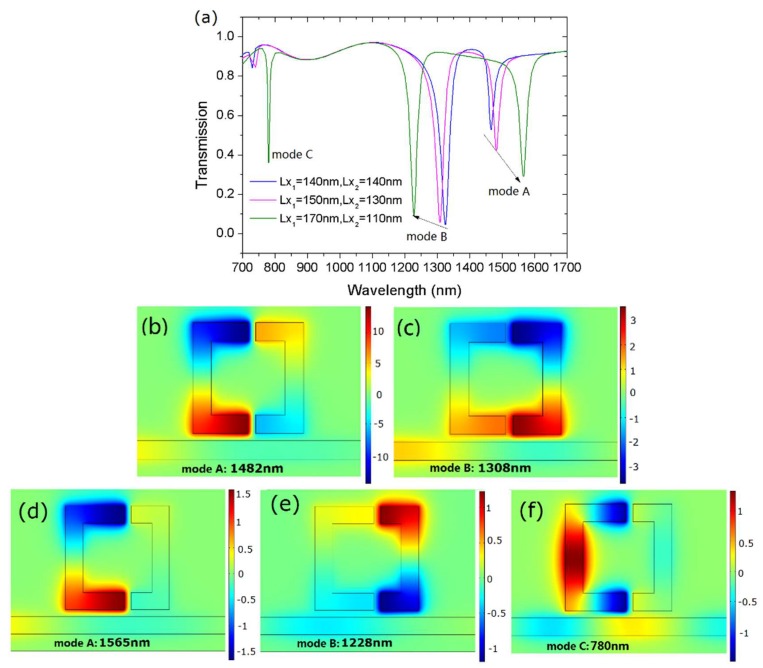
(**a**) The transmission spectra at different arm lengths *Lx*_1_ and *Lx*_2_. *H_z_* field patterns at resonance wavelengths when (**b**,**c**) *Lx*_1_ = 150 nm, *Lx*_2_ = 130 nm, and (**d**–**f**) *Lx*_1_ = 170 nm, *Lx*_2_ = 110 nm. The gap width is *g* = 20 nm.

Consider two side-coupled resonators of the same dimensions. The arm length of U-shaped resonators *Lx*_1_ = *Lx*_2_ = 140 nm. The width of the two resonators are *W*_1_ = *W*_2_ = 50 nm. Their resonant frequencies would be equal, namely *ω*_1_ = *ω*_2_. The transmission spectrum was plotted by a blue curve in Figure 2a. The frequency splitting occurred in the case of two U-resonators. The obtained mode splitting was consistent with the theoretical prediction by Equation (4). The corresponding wavelengths were *λ*_−_ = 1325 nm and *λ*_+_ = 1465 nm. Here, we label them mode A and B, respectively. There is a transparency window between the two dips.

The case of different resonator size is also investigated. We fixed the widths and varied the arm length *Lx* such that *ω*_1_ ≠ *ω*_2_. The transmission spectra at different arm lengths is also shown in Figure 2a. Define the difference of arm length as Δ*L* = *Lx*_1_ − *Lx*_2_. It was observed that, with the increase of Δ*L*, the two resonant wavelengths moved in a different direction. Mode A had a red shift, while mode B displayed a blue shift.

To explain the shift of dip wavelength, we plotted the magnetic field distributions. Figure 2b,c displays *H_z_* field distributions at resonance wavelengths of the pink curve. As shown, at the resonance wavelength of 1308 nm, the field distribution appears symmetrical; however, most of the energy is confined in the right resonator. At the resonance wavelength of 1482 nm, the field distribution is anti-symmetrical and most energy is confined in the left resonator. For the green curve, the unequal energy distribution is more obvious, as shown in Figure 2d–f. Therefore, mode A was predominantly influenced by the left resonator, i.e., *Lx*_1_. In contrast, mode B was predominantly influence by the right resonator, i.e., *Lx*_2_. From Equation (1), it is known that the resonance wavelength is proportional to the cavity length. From the blue line to the green line, the arm length *Lx*_1_ increases while *Lx*_2_ decreases; accordingly, the resonance wavelength *λ*_A_ shifts to long wavelength and *λ*_B_ shifts to short wavelength.

Note that the green line in Figure 2a has one additional transmission dip than other two lines. We define it as mode C. The corresponding resonance wavelength was 780 nm. To examine the physical mechanism of this dip, its *H_z_* field pattern is given by Figure 2f. The field distribution shows it is a higher-order mode. In addition, most energy was confined in the left resonator, which means this mode could have been influenced by *Lx*_1_.

**Figure 3:**
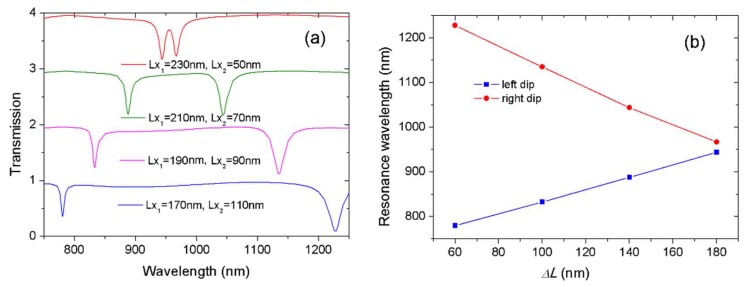
(**a**) The transmission spectra with different cavity arm lengths *Lx*_1_ and *Lx*_2_; (**b**) two resonance wavelengths vs. the difference of cavity lengths Δ*L* = (*Lx*_1_ − *Lx*_2_).

Inspired by the various shifting tendencies of these modes, we further enlarged the difference of cavity length Δ*L* to investigate the effects of structural parameters on resonance frequencies. We set *Lx*_1_ = 140 + Δ*L*/2 and *Lx*_2_ = 140 − Δ*L*/2. Figure 3a shows the evolution of transmission spectra for modes B and C at different arm lengths. From bottom to top, two dips moved towards each other as the difference of cavity length Δ*L* increased from 60 to 180 nm. The previous results have shown that mode C originated from a high-order mode and its resonance wavelength was largely determined by the left resonator, i.e., *Lx*_1_. In contrast, mode B stemmed from a low-order mode and its resonance wavelength was highly influenced by the right resonator, i.e., *Lx*_2_. Since *Lx*_1_ increased and *Lx*_2_ decreased from bottom to top, the left dips shifted to a long wavelength and the right dips shifted to short wavelength. Two transmission dips became increasingly close. When Δ*L* = 180 nm, there was a very narrow transparency window in the transmission spectrum, as displayed by the red line. This differed from the transparency window depicted by the blue curve in Figure 2a; the spectral response in Figure 3a resulted from the hybridized resonances with detuned frequencies and different orders.

The dip wavelength as a function of the length difference Δ*L* is shown in Figure 3b. Both dips had a linear relationship with the variation of cavity length. The results agreed well with the theoretical analysis of Equation (1). With the increase of Δ*L*, the difference of resonance wavelengths diminished and the transparency window narrowed.

Figure 3 demonstrates that the controllable resonance wavelength was realized by changing the resonator length. Two U-shaped resonators could be obtained by inserting two metal blocks in a rectangular cavity. The width of the metal blocks is the coupling distance *g*. We slid metal blocks to conveniently change the arm length *Lx*_1_ and *Lx*_2_. Accordingly, the proposed design of the U-shaped resonator had the advantage of structural flexibility.

**Figure 4:**
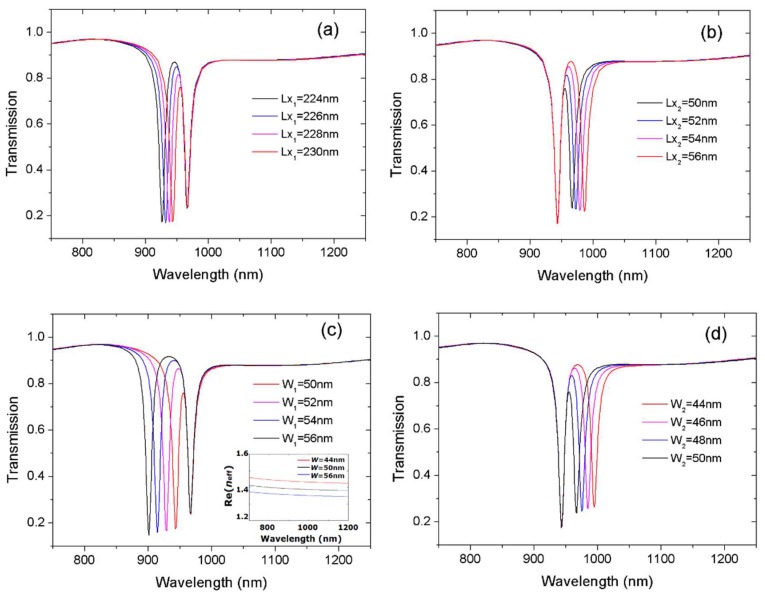
The transmission spectra at different length and width of cavities. The variable is (**a**) *Lx*_1_; (**b**) *Lx*_2_; (**c**) *W*_1_; (**d**) *W*_2_. The inset in (**c**) shows the effective index *n*_eff_ versus wavelength at different width *W*. One dip was unchanged and the other dip shifted towards a long wavelength with increased cavity length in (**a**,**b**) or shifted towards a short wavelength with increased cavity width in (**c**,**d**).

To further clarify the influence of each cavity on resonant wavelengths, we fixed the structural parameters of one cavity while varying the parameters of another cavity. The initial parameters were *Lx*_1_ = 230 nm, *Lx*_2_ = 50 nm, and *W*_1_ = *W*_2_ = 50 nm. The coupling distance *g* = 20 nm. Figure 4a,b present the transmission spectra with varied cavity length *Lx*_1_ and *Lx*_2_. Notably, the left transmission dip shifted from 944 to 926 nm as *Lx*_1_ increased from 224 to 230 nm. Meanwhile, the right dip was unchanged, as shown in Figure 4a. When *Lx*_2_ increased from 50 to 56 nm, the right transmission dip shifted from 967 to 986 nm. Simultaneously, the left dip was unchanged. Figure 4a,b shows the redshift for one dip with an unchanged second dip with increased cavity length. 

The influence of resonator width on the resonance wavelength was also investigated. Figure 4c,d present the transmission spectra with variable *W*_1_ and *W*_2_. Notably, the left transmission dip shifted from 926 to 901 nm as *W*_1_ increased from 50 to 56 nm. However, the right dip remained unchanged. When *W*_2_ decreased from 50 to 44 nm, the right transmission dip shifted from 967 to 995 nm, and the left dip was unchanged. These results were consistent with the theoretical prediction. It is known from Equation (1) the resonance wavelength depends on the effective index *n*_eff._ Dispersion equation shows *n*_eff_ can be determined by the width of the MDM waveguide. The inset in Figure 4c shows the real part of the effective index Re(*n*_eff_) versus the wavelength at different waveguide width, *W*. The bigger the width *W* was, the smaller the effective index Re(*n*_eff_) was. As a result, there was blueshift for resonance wavelength which corresponded to an increase of cavity width.

Figure 4 demonstrates that the transmission dips can be separately controlled. Both the width and the length of resonators could tune resonance wavelengths. Independently tunable resonance was realized by the use of two U-shaped resonators. The two resonances had different responses to the variations of the structural dimensions because of their different physical origins. One resonator contributed to the shift of the left dip while the other contributed to the shift of the right dip. Therefore, the precise control of operating wavelength of resonance was achievable only by changing the structural parameters. Because the control on double resonances was independent, it may have important applications in multi-parameter sensing.

### 3.2. Self-Reference Sensing

**Figure 5:**
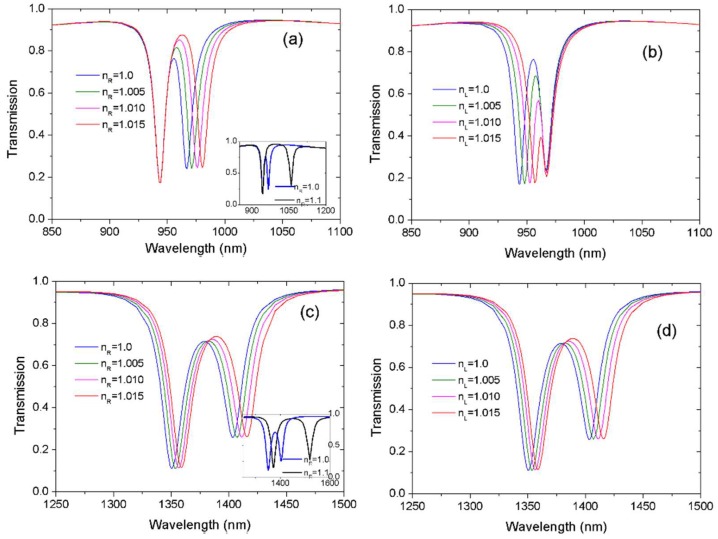
Transmission spectra with different refractive index *n.* Only one cavity is filled with sensing medium. (**a**,**b**) The arm length *Lx*_1_ = 230 nm, *Lx*_2_ = 50 nm, and gap width *g* = 20 nm. (**c**,**d**) The arm length *Lx*_1_ = 140 nm, *Lx*_2_ = 140 nm, and gap width *g* = 40 nm. The insets in (**a**,**c**) show the transmission at large index *n*_R_ = 1.1; the result of *n*_R_ = 1.0 is also shown.

On the basis of the proposed waveguide-cavity system, we investigate the effect of variation of the refractive index (*n*) on the double resonances. Only one cavity is filled with sensing medium. Figure 5a illustrates the transmission spectra with different refractive index *n*_R_ filled in the right cavity. The arm length for *Lx*_1_ = 230 nm and for *Lx*_2_ = 50 nm. Refractive index increased from 1 to 1.015 at intervals of 0.005. When *n*_R_ = 1.0, the transmission spectrum corresponded to the red line in Figure 4a. It could be observed that the peak and the right dip exhibited a red shift with the increase of *n*_R_. The inset in Figure 5a shows the transmission at *n*_R_ = 1.1. In the case of large index variation, the red shift of the right dip was significantly more noticeable. In contrast, the left dip was always unchanged because almost all the energy was concentrated in the right resonator, making the resonance related to the right resonator more sensitive to the index variations. When left cavity was filled with sensing medium, the dependence of transmission spectra on index variation *n*_L_ were also studied. The result is shown in Figure 5b. Only the left dip shifted to a long wavelength with the increase of index *n*_L_ while the right dip remained unchanged. Figure 5a,b reveal that the two resonances had different responses to the refraction index variation in the resonator. The unique feature provided an excellent scheme for nanoscale self-reference sensing. In Figure 5a, the changed right dip could be used for sensing, and the fixed left dip provided a reference signal. By monitoring the left resonance, which was no influenced by index variations, one could determine to what extent the intensity fluctuations and local temperature affected the wavelength shift. As a result, the influences of external fluctuations could be excluded or reduced. This may be of particular importance for achieving accurate sensing in unstable and complicated environments.

By comparison, the effect of the variation of the refractive index on mode A and B was investigated. The parameters were same as the blue curve in Figure 2a apart from the coupling distance. *Lx*_1_ = 140 nm, *Lx*_2_ = 140 nm and *g* = 40 nm. Figure 5c shows the transmission spectra with different refractive index *n*_R_ filled in the right cavity. In contrast to Figure 5a, both dips shifted to a long wavelength with the increase of *n*_R_. This is a commonly reported property [13,14,15]. The inset in Figure 5c shows the red shift was more noticeable at larger *n*_R_ = 1.1. When only the left cavity was filled with sensing medium, the evolution of the transmission at different refractive index *n*_L_ is shown in Figure 5d. Both dips exhibited red shift with the increase of *n*_L_. In comparison, double resonances in Figure 5a,b were independent of each other. The position of the right and left transmission dip depended on the refractive index of dielectric in the right and left cavity, respectively. The difference between Figure 5a,b and Figure 5c,d was attributable to the different physical origin. The spectral profile in Figure 5a,b was induced by the hybridized resonances with detuned frequencies and different orders. The spectral profile in Figure 5c,d resulted from mode splitting attributable to the strong coupling.

**Figure 6:**
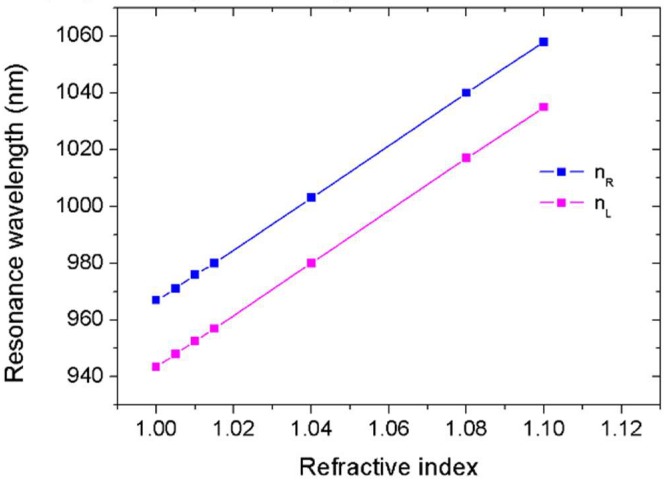
The resonance wavelengths versus refraction index *n*.

To construct a sensor with self-sensing performance, Figure 5a,b presents superior choices. In addition, Figure 5a,b reveals that two transmission dips can be used not only for sensing but also for monitoring. As a result, we flexibly switched one resonance from sensing to monitoring as needed. Figure 5 shows the position of the right/left resonance dip was proportional to the refractive index of the filled medium in the cavity. The relationship between resonance wavelength and refractive index in Figure 5a,b was analyzed in detail and results are presented by Figure 6. It was observed that resonance dips responded linearly to variations in the refractive index. The sensitivity (nm/RIU) of a sensor is usually defined as the shift in the resonance wavelength per unit variation of refractive index [21]. It is expressed by S = Δλ/Δ*n*. The obtained sensitivities for right and left resonance were approximately 917 nm/RIU and 914 nm/RIU, which was excellent compared with those of reported plasmonic sensors [8,9,10,22,23]. Previous research has revealed that the transparency window can be flexibly controlled by structural parameters [24]. Accordingly, the sensing performance of our sensor can be further improved by optimizing structural parameters.

The figure of merit (FOM) is another key parameter for sensors. FOM is defined as S/FWHM [25]. FOM takes into account simultaneous importance of sensitivity and width of the resonance spectrum. The FOM of the proposed system was equal to 180 and 100 RIU^−1^, respectively. The FOM of the sensor showed a significant improvement with respect to the Ref. [23,26]. Through the use of the independent double resonances, the high-performance sensor was demonstrated.

## 4. Conclusions

We have demonstrated independent control on double resonances through a simple MDM waveguide-resonator nanostructure. The transmission dips showed varying responses to the variations of the length and width of the two U-shaped cavities. Accordingly, precise control of operating wavelength of resonance was available only through a change in the structural parameters of the cavities. More importantly, two resonance dips showed contrasting dependences on the refractive index variations in the individual resonator. Accordingly, the designed structure can perform as a refractive index sensor. Because one dip was extremely sensitive to the refraction index while the other dip was independent of the variation of index, the proposed structure may serve as a self-reference sensor by which detection accuracy can be improved. The obtained nanosensor exhibited an excellent sensing performance with a high sensitivity of 917 nm/RIU and FOM of 180 RIU^−1^. This type of plasmonic sensor has important practical applications in the fields of integrated nanosensing, such as accurate sensing in unstable environments as well as multi-parameter sensing.

## Abbreviations

SPPs	Surface plasmon polaritons
MDM	Metal-dielectric-metal
CMT	Coupled mode theory
FOM	Figure of merit
RIU	Refractive index unit

## References

[1] Roh S., Chung T., Lee B. (2011). Overview of the characteristics of micro- and nano-structured surface plasmon resonance sensors. Sensors.

[2] Tong L., Wei H., Zhang S., Xu H. (2014). Recent advances in plasmonic sensors. Sensors.

[3] Ozbay E. (2006). Plasmonics: Merging photonics and electronics at nanoscale dimensions. Science.

[4] Barnes W.L., Dereux A., Ebbesen T.W. (2003). Surface plasmon subwavelength optics. Nature.

[5] Xie Y., Huang Y., Xu W., Zhao W., He C. (2016). A plasmonic temperature-sensing structure based on dual laterally side-coupled hexagonal cavities. Sensors.

[6] Brolo A.G. (2012). Plasmonics for future biosensors. Nat. Photonics.

[7] Lee K.-L., Huang J.-B., Chang J.-W., Wu S.-H., Wei P.-K. (2015). Ultrasensitive biosensors using enhanced fano resonances in capped gold nanoslit arrays. Sci. Rep..

[8] Lu H., Liu X., Mao D., Wang G. (2012). Plasmonic nanosensor based on fano resonance in waveguide-coupled resonators. Opt. Lett..

[9] Chen J., Sun C., Gong Q. (2014). Fano resonances in a single defect nanocavity coupled with a plasmonic waveguide. Opt. Lett..

[10] Zhao X., Zhang Z., Yan S. (2017). Tunable fano resonance in asymmetric mim waveguide structure. Sensors.

[11] Tang Y., Zhang Z., Wang R., Hai Z., Xue C., Zhang W., Yan S. (2017). Refractive index sensor based on fano resonances in metal-insulator-metal waveguides coupled with resonators. Sensors.

[12] Qiu P., Qiu W., Lin Z., Chen H., Ren J., Wang J.-X., Kan Q., Pan J.-Q. (2017). Dynamically tunable plasmon-induced transparency in on-chip graphene-based asymmetrical nanocavity-coupled waveguide system. Nanoscale Res. Lett..

[13] Qi J., Chen Z., Chen J., Li Y., Qiang W., Xu J., Sun Q. (2014). Independently tunable double fano resonances in asymmetric mim waveguide structure. Opt. Express.

[14] Wu T., Liu Y., Yu Z., Ye H., Shu C., Peng Y., Wang J., He H. (2015). Tuning the fano resonances in a single defect nanocavity coupled with a plasmonic waveguide for sensing applications. Mod. Phys. Lett. B.

[15] Li C., Li S., Wang Y., Jiao R., Wang L., Yu L. (2017). Multiple fano resonances based on plasmonic resonator system with end-coupled cavities for high-performance nanosensor. IEEE Photonics J..

[16] Wang Y., Sun C., Li H., Gong Q., Chen J. (2017). Self-reference plasmonic sensors based on double fano resonances. Nanoscale.

[17] Han Z., Forsberg E., He S. (2007). Surface plasmon bragg gratings formed in metal-insulator-metal waveguides. IEEE Photonics Technol. Lett..

[18] Dionne J.A., Sweatlock L.A., Atwater H.A., Polman A. (2006). Plasmon slot waveguides: Towards chip-scale propagation with subwavelength-scale localization. Phys. Rev. B.

[19] Lu H., Liu X., Mao D. (2012). Plasmonic analog of electromagnetically induced transparency in multi-nanoresonator-coupled waveguide systems. Phys. Rev. A.

[20] Zhang Z.D., Wang R.B., Zhang Z.Y., Tang J., Zhang W.D., Xue C.Y., Yan S.B. (2017). Electromagnetically induced transparency and refractive index sensing for a plasmonic waveguide with a stub coupled ring resonator. Plasmonics.

[21] Liu N., Mesch M., Weiss T., Hentschel M., Giessen H. (2010). Infrared perfect absorber and its application as plasmonic sensor. Nano Lett..

[22] Liu N., Weiss T., Mesch M., Langguth L., Eigenthaler U., Hirscher M., Soennichsen C., Giessen H. (2010). Planar metamaterial analogue of electromagnetically induced transparency for plasmonic sensing. Nano Lett..

[23] Shahamat Y., Vahedi M. (2017). Pump-tuned plasmon-induced transparency for sensing and switching applications. Opt. Commun..

[24] Ren X., Ren K., Cai Y. (2017). Tunable compact nanosensor based on fano resonance in a plasmonic waveguide system. Appl. Opt..

[25] Sherry L.J., Chang S.H., Schatz G.C., Van Duyne R.P., Wiley B.J., Xia Y.N. (2005). Localized surface plasmon resonance spectroscopy of single silver nanocubes. Nano Lett..

[26] Li B., Li H., Zeng L., Zhan S., He Z., Chen Z., Xu H. (2016). Theoretical analysis and applications in inverse t-shape structure. J. Opt. Soc. Am. A Opt. Image Sci. Vision.

